# Primary glomangiosarcoma of the lung: A case report

**DOI:** 10.1186/1749-8090-5-76

**Published:** 2010-10-04

**Authors:** Athanassios Kleontas, Nikolaos Barbetakis, Christos Asteriou, Anastasia Nikolaidou, Aggeliki Baliaka, Ioanna Kokkori, Eleftheria Konstantinou, Anna Grigoriou, Jacob Antzel

**Affiliations:** 1 Cardiothoracic Surgery Department, Theagenio Cancer Hospital, Al. Symeonidi 2, Thessaloniki, 54007, Greece; 2 Pathology Department, Theagenio Cancer Hospital, Al. Symeonidi 2, Thessaloniki, 54007, Greece; 3 Pneumonology - Oncology Department, Theagenio Cancer Hospital, Al. Symeonidi 2, Thessaloniki, 54007, Greece

## Abstract

**Background:**

Glomus tumor is an uncommon neoplasm derived from cells of the neuromyoarterial glomus or glomus body. Most glomus tumours occur in the dermis and subcutaneous tissues. A case of a primary pulmonary glomus tumour originating in the right upper lobe is presented.

**Case presentation:**

A 74-yr-old male was admitted with siccus cough, dyspnea and right-sided chest pain. Computed tomography of the thorax revealed a 4 cm growth of the right upper lobe. Fiberoptic bronchoscopy demonstrated an endobronchial hypervascular mass causing obstruction of the apical segmental bronchus. Pathology report was consistent with pulmonary glomus tumor. The patient underwent a typical right upper lobectomy with mediastinal lymph node dissection. Twelve months later he is free of disease.

**Conclusion:**

Occasionally glomus tumors can occur in extracutaneous sites such as the gastrointestinal tract, bone, genitourinary system and respiratory tract. Primary pulmonary glomus tumors are very rare (our case is the 19^th ^one presented in the international literature) and are often confused with other solid neoplasms such as carcinoids, hemangiopericytomas and tumors belonging to the family of Ewing's sarcoma/primitive neuroectodermal tumours.

## Introduction

Glomus tumors are neoplasms originating from glomus bodies in the dermis or subcutis of the extremities [1]. Extracutaneous presentations occur but are rare, especially in visceral organs where glomus bodies are sparse or even absent [1]. The exact incidence of glomus tumors is unknown. The probable misdiagnosis of many of these lesions as hemangiomas or venous malformations also makes an accurate assessment of incidence difficult [2,3]. A case of a primary pulmonary glomus tumor originating in the right upper lobe is presented.

## Case presentation

A 74-year-old smoking male patient was referred with a persisting siccus cough, dyspnea and right-sided chest pain. Apart from hypertension, his history was negative.

**Figure 1:**
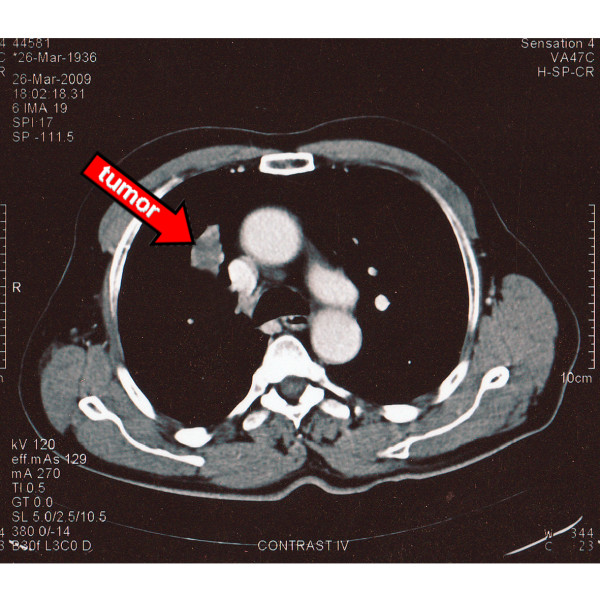
**Chest computed tomography (CT) showed a tumor in the parahilar region of the right upper lobe**.

**Figure 2:**
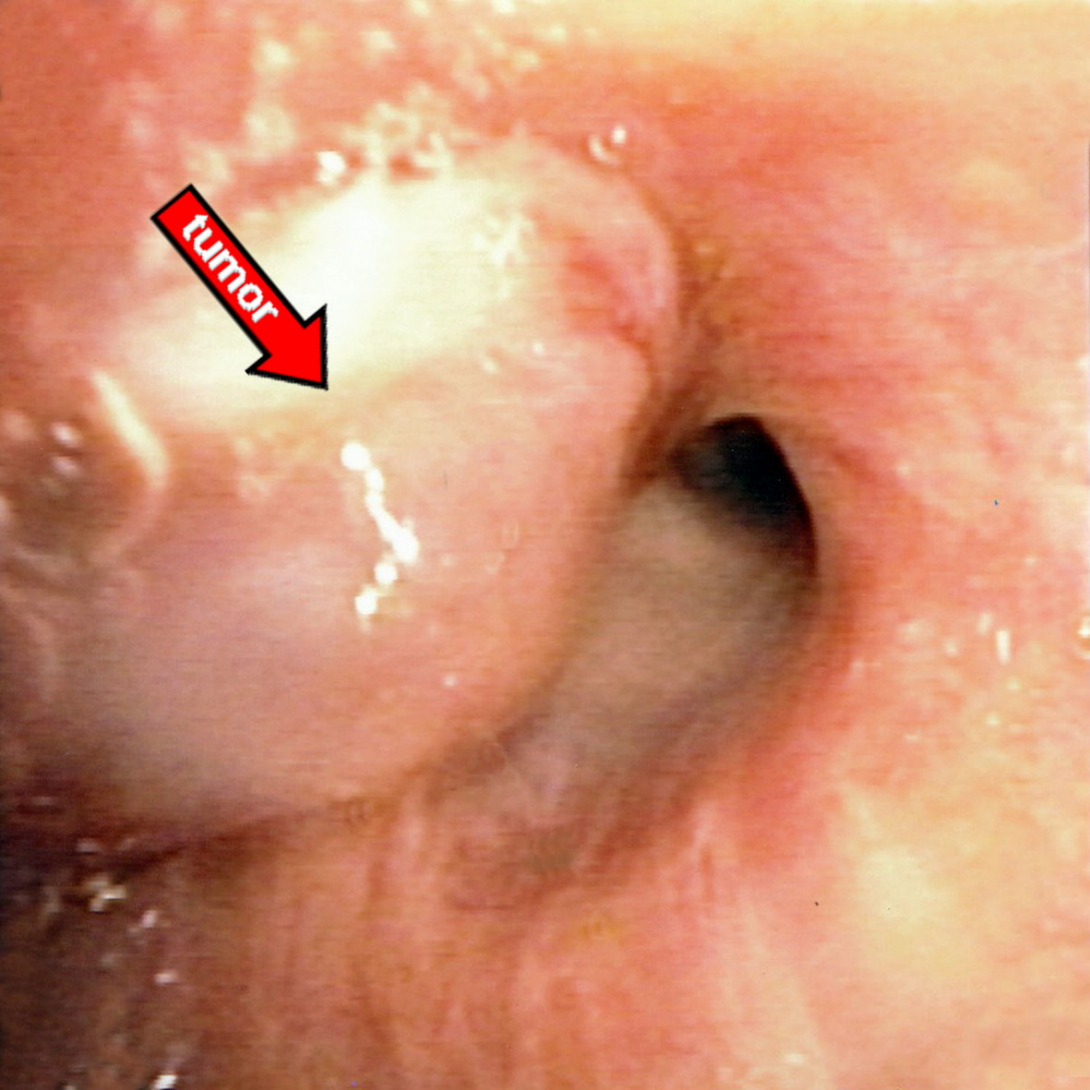
**Fiberoptic bronchoscopy demonstrated an endobronchial hypervascular mass causing obstruction of the apical segmental bronchus**.

Physical examination and routine laboratory tests were normal. Chest x-ray revealed a right upper lobe growth. Chest computed tomography (CT) showed a tumor without inlying calcifications in the parahilar region of the right upper lobe, with a size of 4.0 × 2.6 cm (Figure 1). Positron emission tomographic (PET) scanning showed a low to moderate isotope uptake. No other lesions were detected. Fiberoptic bronchoscopy demonstrated an endobronchial hypervascular mass causing obstruction of the apical segmental bronchus (Figure 2). Pathology report was consistent with pulmonary glomus tumor.

The patient underwent a right mucle-sparing anterolateral thoracotomy and a right upper lobectomy with mediastinal lymph node dissection.

**Figure 3:**
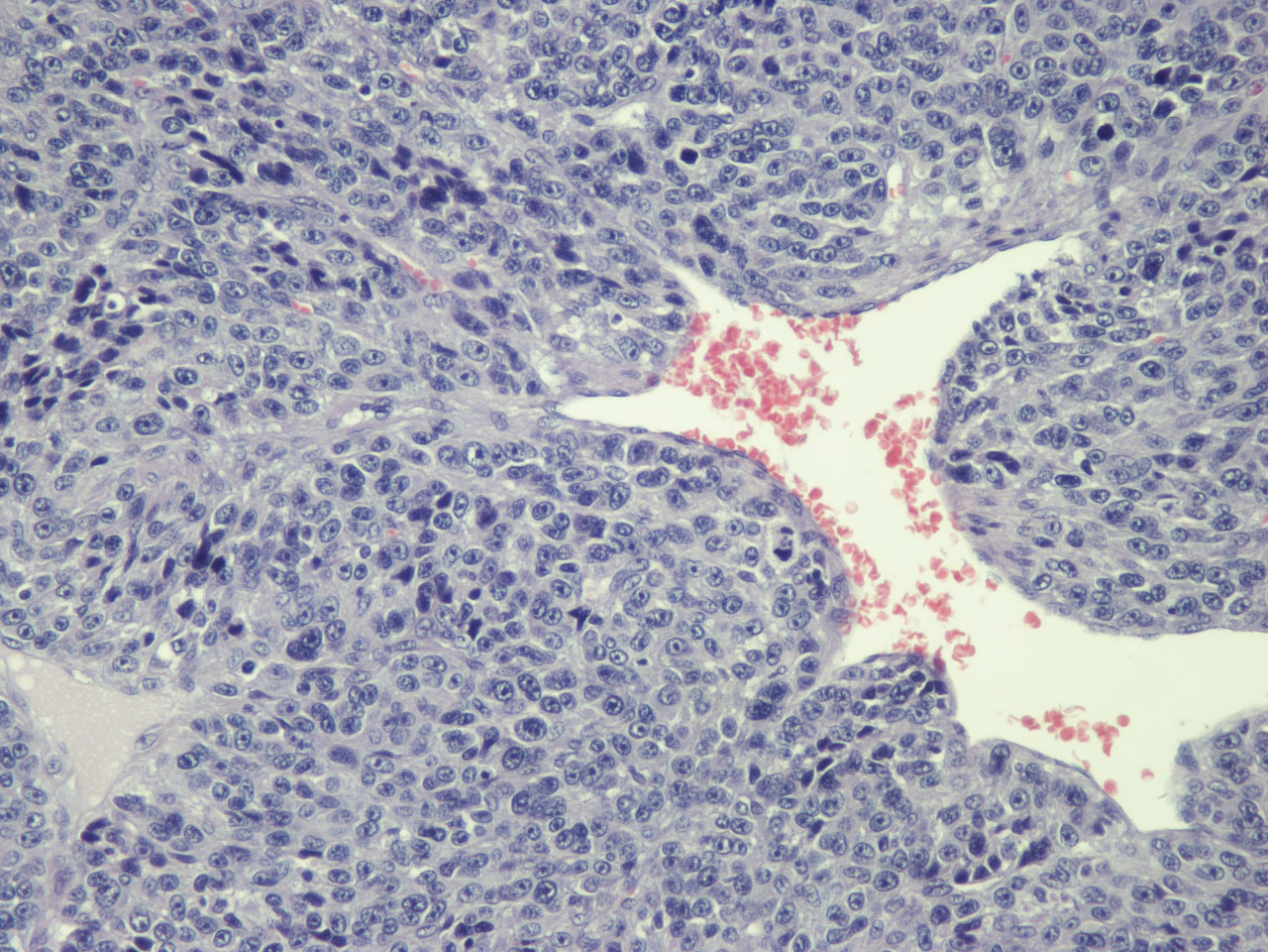
**The tumor was encapsulated and was composed of sheets and nests of small, uniform, rounded cells with centrally placed round nuclei (H-E × 200)**.

**Figure 4:**
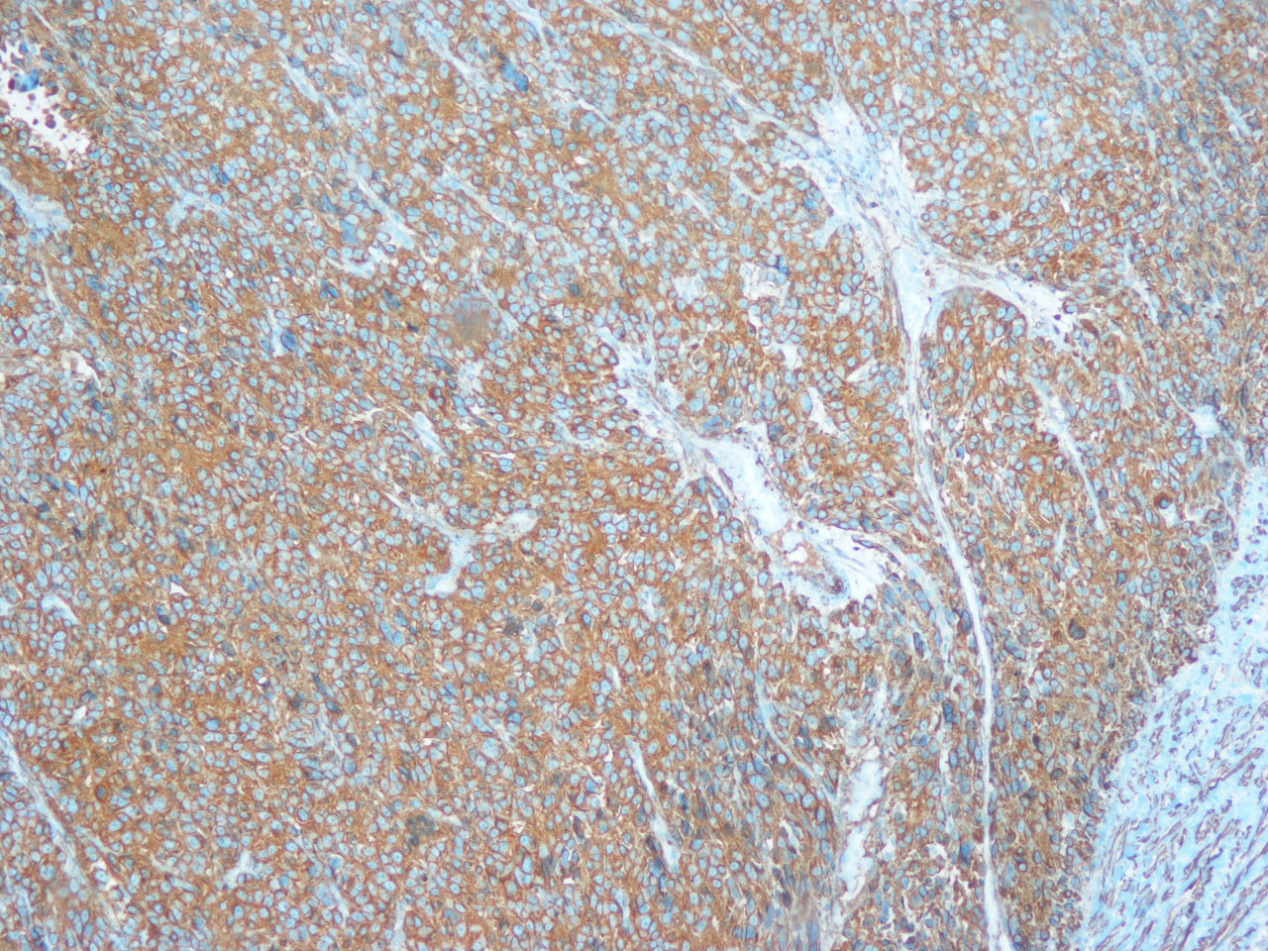
**Immunohistochemistry: tumor cells were positive for smooth muscle actin (SMA × 100)**.

**Figure 5:**
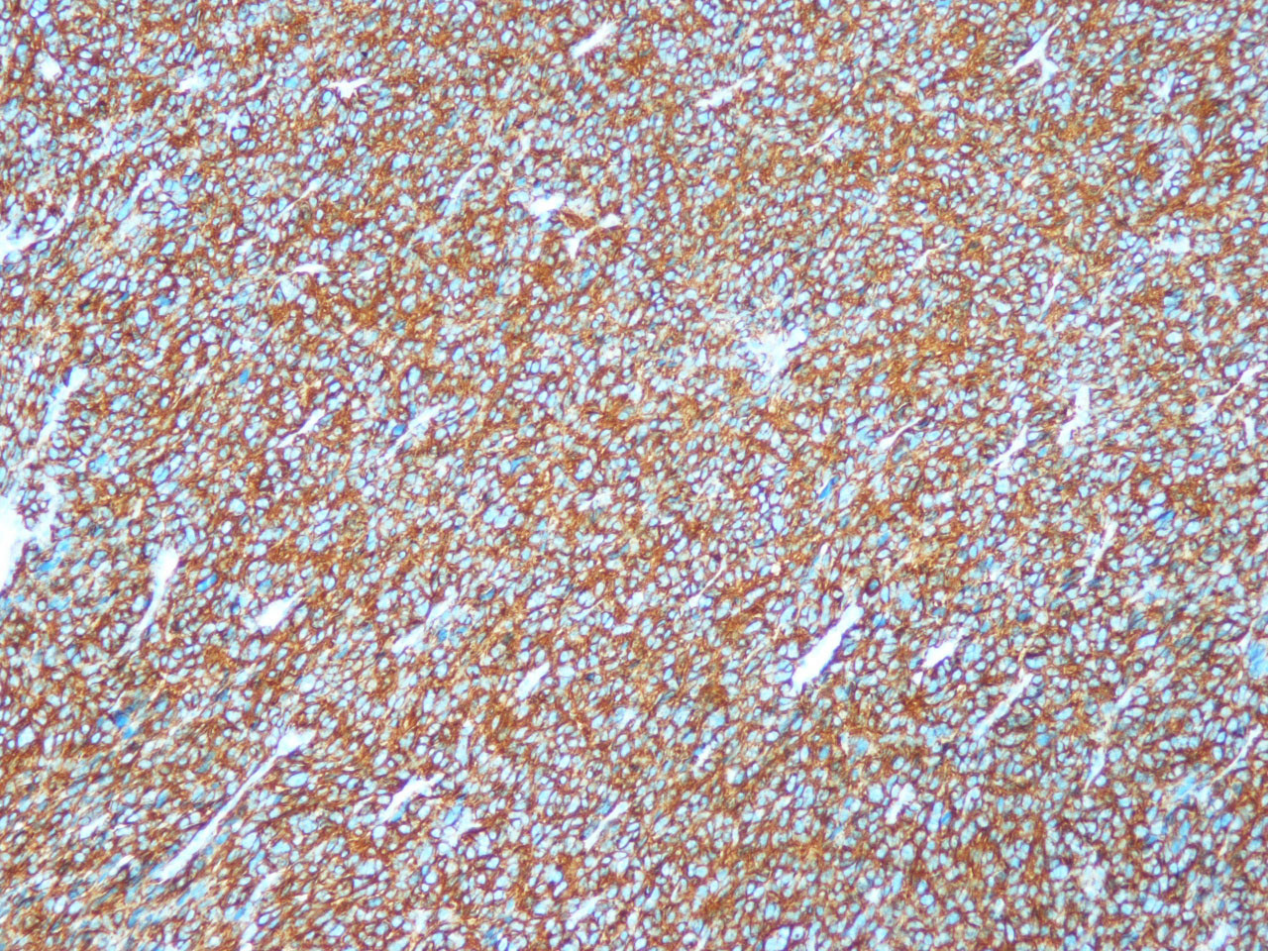
**Immunohistochemistry: tumor cells were positive for caldesmon (× 100)**.

**Figure 6:**
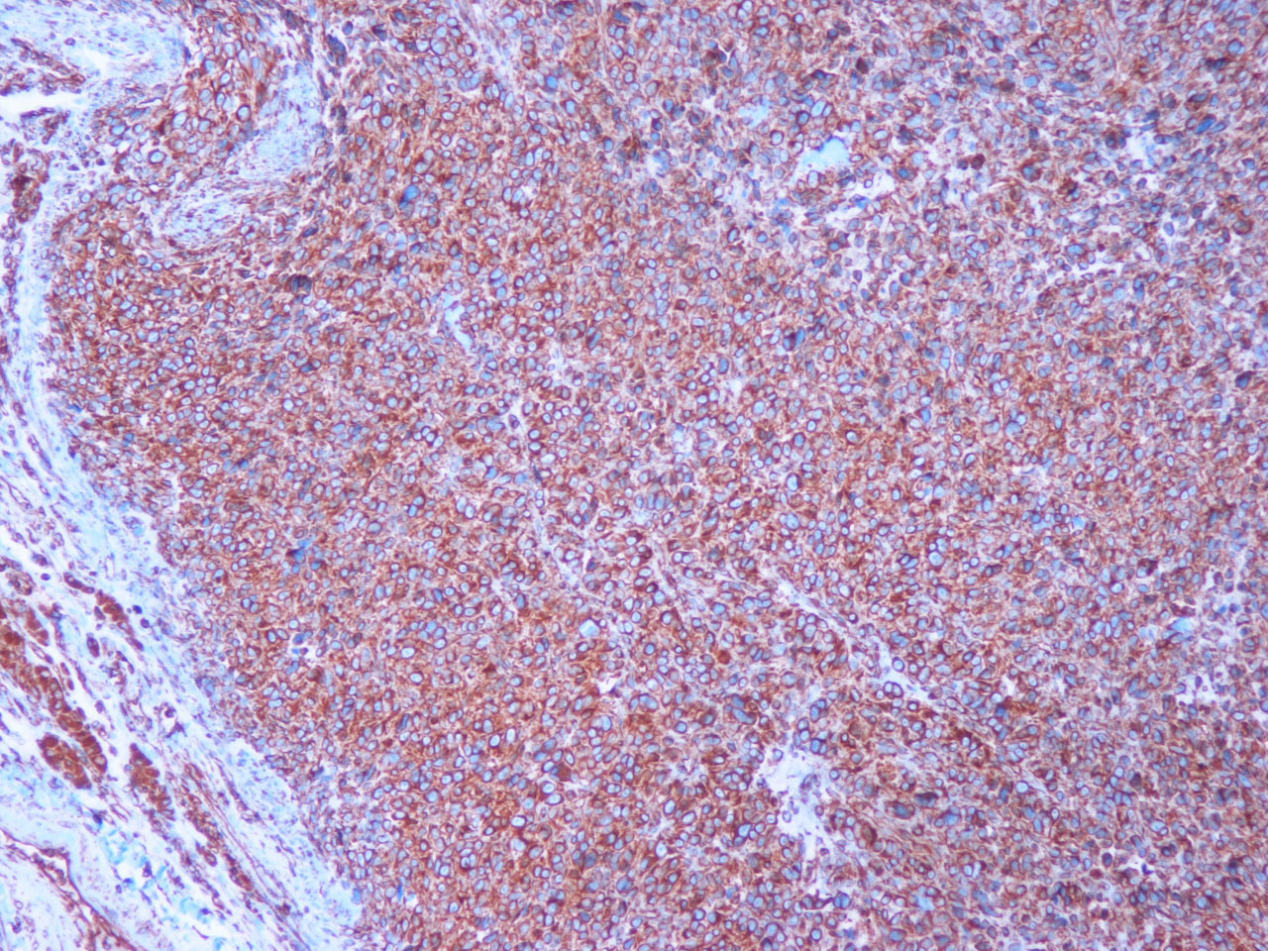
**Immunohistochemistry: tumor cells were positive for vimentin (× 100)**.

Macroscopically, a circumscribed soft mass, measuring 3,4 cm in greatest dimension, with white to pink cut surface was found. Histologically, the tumor was encapsulated and was composed of sheets and nests of small, uniform, rounded cells with centrally placed, round nuclei; amphophilic to lightly eosinophilic cytoplasm and prominent nucleoli (glomus cells) surrounding capillary sized vessels (Figure 3). The presence of nuclear atypia, high mitotic activity (up to 5 m/10 HPF), atypical mitosis and size > 2 cm suggested malignancy. The tumor focally infiltrated the surrounding lung structures but no bronchi or pleura were involved in the tumoral process. Immunohistochemically, tumor cells were positive for smooth muscle actin (SMA) (Figure 4), caldesmon (Figure 5) and vimentin (Figure 6), whereas they were negative for CD56, chromogranin, cytokeratin proteins, desmin, p63 protein and TTF-1. The final pathological diagnosis was intrapulmonary malignant glomus tumor (glomangiosarcoma), round cell type.

The patient made an uneventful recovery. Twelve months later he is free of disease.

## Discussion

Solitary glomus tumors are more frequent in adults than in others. Multiple glomus tumors develop 10-15 years earlier than single lesions; about one third of the cases of multiple tumors occur in those younger than 20 years. Congenital glomus tumors are rare; they are plaquelike in appearance and are considered a variant of multiple glomus tumors.

Glomus tumours can be subdivided pathologically into glomus tumour proper, glomangioma and glomangiomyoma, based on the relative predominance of the three major constituents: round glomus cells in glomus tumour proper; blood vessels in glomangioma; and spindle cells in glomangiomyoma. Glomus tumour proper is the most common, followed by glomangioma. Glomangiomyoma is the rarest variant with a frequency as low as 8% of all glomus tumours [4]. Glomus tumors are highly vascular, and are usually solitary, caused by a proliferation of glomus cells, which make up a portion of the glomus body. Because they are usually benign and slow-growing, mortality rates are low (less than 15 percent). However, their growth can cause significant damage to surrounding tissue.

The differential diagnosis consists of a wide variety of neoplasms, most notably: carcinoid tumor, hemangiopericytoma, paraganglioma, smooth muscle neoplasms and metastatic tumors [5]. Carcinoid tumors are most commonly confused with glomus tumors, since they possess a similar cytological appearance. In spite of this, they were excluded because of the absence of the somewhat typical coarsely granular to salt-and-pepper chromatin - in contrast to the finer chromatin pattern of glomus tumors - and the negative staining for neuroendocrine markers [6]. Hemangiopericytoma is another rare tumor that should be considered. Nevertheless, a glomus tumor differs because of its round epithelioid cells and regular oval to round nuclei, whereas hemangiopericytomata consist of more polygonal to spindle-shaped cells with elongated nuclei. Although spindle cells were found in the present case as well, their low quantity and focal distribution were not very suggestive for hemangiopericytoma. Moreover, the ramifying to staghorn vasculature pattern, which is archetypical for hemangiopericytoma, was absent [7]. Paraganglioma, on the other hand, could be excluded because of the absence of sustentacular cells and the typical 'Zellballen' pattern, combined with the negative staining for neuroendocrine markers [8]. Other neoplasms, such as smooth muscle tumors and secondary metastatic lesions have distinctive histological and immunohistochemical features and were effortlessly differentiated from glomus tumors.

## Conclusions

Despite that intrapulmonary glomus tumors are generally benign neoplasms, four malignant cases have been described so far, with the present case to be the 5^th ^one. Complete surgical excision is the treatment of choice with excellent prognosis [9-11].

## Consent

Written informed consent was obtained from the patient for publication of this case report and accompanying images. A copy of the written consent is available for review by the Editor-in-Chief of this journal.

## Competing interests

The authors declare that they have no competing interests.

## Authors' contributions

Authors' contributions AK, NB, CA, IK, EK, AG and JA took part in the care of the patient and contributed equally in carrying out the medical literature search and preparation of the manuscript. AN and AB were responsible for the pathology report. All authors approved the final manuscript.
